# Plasma Proteomics of Type 2 Diabetes, Hypertension, and Co-Existing Diabetes/Hypertension in Thai Adults

**DOI:** 10.3390/life14101269

**Published:** 2024-10-05

**Authors:** Puriwat Fakfum, Hataichanok Chuljerm, Wason Parklak, Sittiruk Roytrakul, Narumon Phaonakrop, Peerasak Lerttrakarnnon, Kanokwan Kulprachakarn

**Affiliations:** 1 Research Institute for Health Sciences, Chiang Mai University, Chiang Mai 50200, Thailand; nuengpuriwat@gmail.com (P.F.); hataichanok.ch@cmu.ac.th (H.C.); wason.p@cmu.ac.th (W.P.); 2 National Center for Genetic Engineering and Biotechnology (BIOTEC), National Science and Technology Development Agency, Pathum Thani 12120, Thailand; sittiruk@biotec.or.th (S.R.); narumon.pha@biotec.or.th (N.P.); 3 Aging and Aging Palliative Care Research Cluster, Department of Family Medicine, Faculty of Medicine, Chiang Mai University, Chiang Mai 50200, Thailand

**Keywords:** proteomics, type 2 diabetes, hypertension, aging

## Abstract

The study explored proteomics to better understand the relationship between type 2 diabetes (T2DM) and hypertension (HT) in Thai adults, using shotgun proteomics and bioinformatics analysis. Plasma samples were taken from 61 subjects: 14 healthy subjects (mean age = 40.85 ± 7.12), 13 with T2DM (mean age = 57.38 ± 6.03), 16 with HT (mean age = 66.87 ± 10.09), and 18 with coexisting T2DM/HT (mean age = 58.22 ± 10.65). Proteins were identified using liquid chromatography-tandem mass spectrometry (LC-MS/MS). Protein–protein interactions were analyzed using the Search Tool for the Retrieval of Interacting Genes/Proteins (STRING) version 11.5. We identified six unique proteins in T2DM patients, including translationally controlled 1 (TPT1) and nibrin (NBN), which are associated with the DNA damage response. In HT patients, seven unique proteins were identified, among them long-chain fatty acid-CoA ligase (ASCL), which functions in the stimulation of triacylglycerol and cholesterol synthesis, and NADPH oxidase activator 1 (NOXA1), which is involved in high blood pressure via angiotensin II-induced reactive oxygen species (ROS)-generating systems. In coexisting T2DM/HT patients, six unique proteins were identified, of which two—microtubule-associated protein 1A (MAP1A)—might be involved in dementia via RhoB-p53 and diacylglycerol kinase beta (DGKB), associated with lipid metabolism. This study identified new candidate proteins that are possibly involved in the pathology of these diseases.

## 1. Introduction

Diabetes mellitus (DM) is a chronic metabolic disease characterized by a high blood sugar level due to impairment in either insulin secretion, insulin action, or both. Persistent hyperglycemia contributes to long-term damage, dysfunction, and failure of various organs and tissues, especially the eyes, vascular system, kidneys, nerves, and heart [1,2]. HT is a major cardiovascular risk factor that often accompanies both type 1 and type 2 diabetes mellitus. According to the World Health Organization (WHO), HT is up to twice as common in people with diabetes as in the general population [3,4]. In white Europeans, 10–30% of subjects with type 1 diabetes and 60–80% of those with newly diagnosed T2DM were found to be hypertensive [5]. Probable causes of the increase in T2DM incidence include a rising prevalence of obesity and longer survival for older people. Most people with T2DM are insulin resistant, as are approximately half of those with essential HT. Furthermore, research indicates that HT and DM share common pathways such as obesity, inflammation, and oxidative stress [6].

Proteomics has emerged as an important tool in several different fields of medical research for unraveling underlying biochemical mechanisms, assessment of response to treatment, and early disease detection. Proteomic studies of diabetes and other complications in cells, tissues, and fluids have attempted to identify the proteins involved, which might be potential therapeutic targets for the treatment or prevention of T2DM with complications, as well as to understand the biochemical mechanisms involved. Investigation of the expression of proteins in the serum of people with T2DM revealed that apolipoprotein C3, transthyretin, apolipoprotein E, leptin, and C reactive protein (CRP) were increased and that albumin, transferrin, and apolipoprotein A-I were decreased in diabetic patients compared to controls. The discovery of these proteins may help explain the pathogenesis of T2DM and enable early detection of risk for this disease and its complications [7,8]. A proteomic analysis of the plasma proteome in people with T2DM and nephropathy found 31 different proteins. Most of these proteins help the body adapt its metabolism. The quantitative proteomic method also found new biomarkers, such as choline/ethanolamine kinase and calpain-7 [9], as well as plasma markers of T2DM, such as ficolin-3 and apolipoprotein A-I. Proteome analysis in hypertensive patients with left ventricular diastolic dysfunction revealed a down-regulation of collagen types 1 and 6 but an up-regulation of collagen type 3 fragments [10]. There have been no proteome studies on coexisting T2DM and HT. In this study, shotgun proteomics was used to identify proteins in the plasma as well as to explain the pathological mechanisms that could be associated with aging in Thai adults, which may also be helpful in improving prevention, diagnosis, and treatment.

## 2. Materials and Methods

### 2.1. Sample Collection and Ethical Approval

Human plasma samples were collected from a total of 61 subjects: 14 healthy subjects, 13 with T2DM, 16 with HT, and 18 with coexisting T2DM/HT. The criteria used for diagnosing T2DM and HT were based on guidelines provided by the WHO. All T2DM patients had typical diabetic symptoms as well as a single fasting plasma glucose level of 126 mg/dL (7.0 mmol/L) or higher. All hypertensive patients had blood pressure readings exceeding 140/90 mmHg. Healthy individuals with a fasting blood glucose level below 100 mg/dL (5.5 mM) and blood pressure less than 140/90 mmHg were selected as the control. The study samples presented with no comorbidities. In each group, all patient samples were diagnosed by a physician and treated with medication. T2DM patients were treated with biguanides, and HT patients were treated with angiotensin-converting enzyme inhibitors. This study was approved by the Maharaj Nakorn Chiang Mai Hospital Research Ethics Committee (Research ID: 1755/Study code No. BIO-2556-01755). Written informed consent was obtained from all participating subjects.

### 2.2. One-Dimensional (SDS-PAGE) Gel Electrophoresis and In-Gel Tryptic Digestion

The protein concentration of each serum sample was measured using the Lowry method [11]. We adjusted each sample to the same protein concentration and then pooled them by group, including T2DM, HT, T2DM/HT, and healthy subjects. A pooled plasma sample of 40 μg from each group was loaded into a 12.5% sodium dodecyl sulfate polyacrylamide (SDS, Sigma-Aldrich, Burlington, MA, USA) gel electrophoresis (SDS-PAGE) system. After completion of electrophoresis, protein bands were visualized by silver staining [12]. The protein bands were then divided into small pieces of about 1 mm^3^. The gel pieces were digested by in-gel digestion using the in-house protocol developed by the Proteomics Research Laboratory, Genome Institute, National Center for Genetic Engineering and Biotechnology (BIOTECH), National Science and Technology Development Agency (NSTDA), Thailand [13]. Briefly, each of the gel pieces was dehydrated twice with 200 µL of 100% acetonitrile (ACN, J.T. Baker, Phillipsburg, NJ, USA) for 5 min, then removed and air dried. The proteins in the gel pieces were reduced to break down disulfide bonds with 50 µL of 10 mM dithiothreitol (DTT, USB Corporation, Cleveland, OH, USA) in 10 mM ammonium bicarbonate at room temperature (RT) for 1 h. After removal of the DTT, the proteins were alkylated at RT for 1 h in the dark in the presence of 50 µL of 100 mM iodoacetamide (IAA, GE Healthcare, Chalfont St Giles, Bucks, UK) in 10 mM ammonium bicarbonate (Sigma-Aldrich, USA), after which the IAA was removed. After alkylation, the gel pieces were dehydrated three times with 200 µL of 100% ACN for 5 min each time. The dried gel pieces were then allowed to swell in 20 µL of trypsin (Promega, Madison, WI, USA) solution (10 ng trypsin in 50% ACN/10 mM ammonium bicarbonate), followed by incubation at RT for 20 min. Thirty µL of 30% ACN was added to keep the gels immersed throughout the digestion process. The gels were incubated overnight at RT, and the extracted peptide solution was transferred to a 96-well microplate. To extract peptide digestion products, 30 µL of 50% ACN in 0.1% formic acid (FA, AppliChem, Darmstadt, Germany) was added to the gel plugs and incubated at RT for 10 min in a shaker. The extracted peptides were collected and pooled. The peptide extraction step was performed three times, with 50 µL of 70% ACN in 0.1% FA used to extract the peptides at the final step. The pooled extracted peptides were dried overnight at 40 °C in an incubator and kept at −80 °C for further liquid chromatography-tandem mass spectrometry (LC-MS/MS) analysis.

### 2.3. LC-MS/MS Analysis

LC-MS/MS analysis and associated bioinformatics were performed according to previously described [13,14,15,16,17,18]. The dried tryptic peptides were resuspended in 15 µL of 0.1% formic acid (FA) and then centrifuged at 8161× *g* for 5 min prior to injection. The peptide supernatant was transferred into an insertion tube and injected into an Ultimate 3000 LC system (Dionex, Sunnyvale, CA, USA) coupled to an Electrospray Ionization (ESI)-Ion-TTrap MS. The peptides were then separated in a volume of 100. Eluent A was 0.1% FA, and eluent B was 80% ACN in water containing 0.1% FA. Peptide separation was achieved with a linear gradient from 10% to 70% B for 13 min at a flow rate of 300 nanoliters per minute (nl/min), including a regeneration step at 90% B at 13–15 min and an equilibration step at 10% B, with each run lasting 20 min. Peptide fragment mass spectra were acquired in data-dependent AutoMS mode (2) with a scan range of 300–1500 mass number/charge number (*m*/*z*). Three and up to five precursor ions were selected from the MS scan range of 50–3000 *m*/*z*.

### 2.4. Protein Quantitation Using MS/MS Spectra Differential Analysis 

DeCyder MS 2.0 Differential Analysis software (GE Healthcare, Uppsala, Sweden) [19,20] was used for protein quantitation. The acquired MS/MS raw data were converted to mzXML files using CompassXport 1.3.10 (Bruker, Karlsruhe, Germany) prior to being imported into the DeCyder MS software. The software included two analysis features: peptide detection with the PepDetect module and run-to-run matching with the PepMatch module. The PepDetect module uses new imaging algorithms to consistently and accurately find peptides, remove background information, deconvolve isotopes and charge states, and figure out peak volumes. The software’s visualization tools offer new capabilities for optimizing chromatography and MS/MS settings. In addition, the PepMatch module supports a wide range of experimental designs, such as control/treated experiments as well as time-dose studies. It can detect small quantitative differences between peptides across multiple runs with high statistical confidence (*p* < 0.05).

### 2.5. Protein Identification by Database Searching

The MS/MS data analyzed by DeCyder MS were submitted to a database search using Mascot software version 2.2 (Matrix Science, London, UK) [21]. For protein identification, the analyzed data were searched against the National Center for Biotechnology Information (NCBI) database under 1% false discovery rate (FDR) determination [22]. The search parameters included “Homo sapiens complex as taxonomy” and “trypsin as the enzyme with 1 missed cleavage allowed”. Carbamidomethyl of cysteine was set as the fixed modification, while oxidation of methionine was set as the variable modification, and ESI-TRAP was set as the instrument type. The peptide mass tolerance and the fragment mass tolerance were set at ±1.2 Da and ±0.6 Da, respectively. The group of proteins of interest from the Mascot search results was categorized based on biological processes according to UniProt (http://www.uniprot.org/ accessed on 1 January–31 October 2023). The same procedure was used to search for the molecular weights, molecular functions, and biological processes. 

### 2.6. Protein–Protein Interaction Analysis

Among the unique proteins found in each of the groups were protein–protein interactions identified using STRING 11.5 (http://string-db.org/ accessed on 1 November–30 December 2023). Also found using STITCH 4.0 (http://stitch.embl.de/ accessed on 1 November–30 December 2023) were protein interactions with chemicals and drugs. The data on protein interaction with chemicals and drugs are shown in the Supplementary Materials.

### 2.7. Statistical Analysis

The fasting plasma glucose (mg/dL), age (years), and blood pressure (mmHg) of the healthy group and the patient groups were compared using one-way analysis of variance (ANOVA) and Fisher’s exact test for categorical variables (IBM SPSS, New York, NY, USA, licensed by Chiang Mai University). All statistical analyses were considered statistically significant at *p* < 0.05.

## 3. Results

### 3.1. Clinical Characteristics of Study Samples and Statistical Analysis 

Table 1 shows there were 14 individual plasma samples from the healthy subjects (control), 13 individual samples from T2DM patients, 16 individual samples from HT patients, and 18 individual samples from the coexisting T2DM/HT group. We compared age (years), fasting plasma glucose (mg/dL), and blood pressure (mmHg), as shown in Table 1. The average age of the T2DM, HT, and T2DM/HT patients was significantly higher than that of the healthy subjects. In addition, the fasting plasma glucose of the T2DM and T2DM/HT patients was significantly higher than that of both the healthy subjects and the HT patients. The systolic and diastolic blood pressures of the HT patients were significantly higher than those of the healthy subjects and T2DM patients.

### 3.2. Proteomic Analysis and Protein Identification

**Figure 1:**
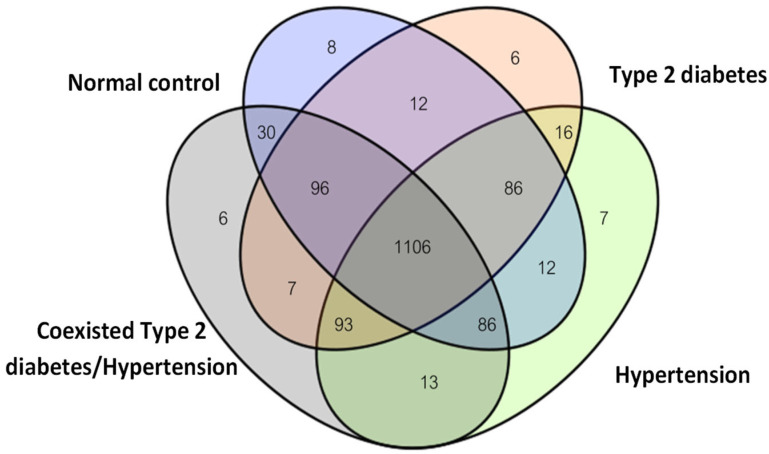
Venn diagram showing the distribution of unique and overlapping plasma proteins in the control patients, T2DM patients, HT patients, and patients with coexisting T2DM/HT.

A total of 1584 differentially expressed proteins were identified in the plasma samples. The numbers of proteins identified in the four groups are summarized in Figure 1. The differential identification of peptides in the healthy control, T2DM, HT, and coexisting T2DM/HT groups totaled 1436, 1422, 1419, and 1437, respectively. The healthy control group had eight unique proteins; the T2DM group had six unique proteins; the HT group had seven unique proteins; and the group suffering from T2DM/HT had six unique proteins. Both T2DM and HT contained sixteen proteins, T2DM and T2DM/HT shared seven proteins, and HT and T2DM/HT shared thirteen proteins (Figure 1).

### 3.3. Functional Categories of Unique Proteins

To investigate the mechanisms underlying T2DM and HT, functional categories of all the unique proteins were searched in Uniprot KB using Homo sapiens as the taxonomy (http://www.uniprot.org/ accessed on 1 January–31 October 2023), as shown in Table 2, Table 3, Table 4 and Table 5.

In the healthy control group, there were eight unique proteins, which were divided into five functional categories, i.e., transcription, signaling, cellular processes, ROS generation, and metabolism (Table 2). The eight proteins are: (1) NF-kappa-B inhibitor epsilon, an NF-kappa-B inhibitor associated with other transcription factors and apoptosis-related proteins involved in the disruption of transcriptional processes; (2) synaptonemal complex protein 3, which is required for normal meiosis during spermatogenesis and for male fertility; (3) HCG2041603, the function of which is still unknown; (4) protein SZT2, which is associated with superoxide dismutase activity; (5) RANBP2, which is involved with the transport factor (Ran-GTP, karyopherin) mediated protein; (6) diphthine methyl ester synthase, which is involved in the pep-tidyl-diphthamide biosynthesis pathway; (7) diacylglycerol kinase zeta, an enzyme that catalyzes the conversion of diacylglycerol (DAG) to phosphatidic acid (PA); and (8) Bax inhibitor 1, which is involved in negative regulation of the apoptotic process.

In the T2DM group, six unique proteins were found, which can be divided into two functional groups: binding proteins and signaling proteins (Table 3). Uniprot identified the functions of these proteins, which can be characterized as follows: (1) HCG32984, a protein whose function is still unknown; (2) Huntingtin-interacting protein 1-related protein (HIP1R) is related to the apoptotic process, clathrin-mediated endocytosis, and receptor-mediated endocytosis; (3) NBN is a component of the MRE11-RAD50-NBN (MRN complex), which plays a critical role in cellular response to DNA damage; (4) TPT1 protein is a negative regulator of the intrinsic apoptotic signaling pathway that responds to DNA damage; (5) immunoglobulin alpha heavy chain variable region; and (6) immunoglobulin gamma 1 heavy chain variable region. Uniport searches yielded no functional information about these two proteins. The unique proteins identified in the plasma of the type 2 diabetic patient group were determined to be involved with DNA damage.

In patients with HT, seven unique proteins were found and were divided into five functional groups: transcription, signaling, metabolism, protein binding, and protein generation of superoxide (Table 4). The functions of these proteins, as determined using Uniprot, are as follows: (1) SH3 and cysteine rich domain-containing protein functions as a cellular response to heat and in intracellular signal transduction; (2) the protein FAN (factor associated with neutral sphingomyelinase activation) is important for TNF-α induced neutral sphingomyelinase activation factor; (3) citron plays an important role in the regulation of cytokinesis and the development of the central nervous system; (4) ribonuclease 11 precursor plays a role in nucleic acid phosphodiester bond hydrolysis; (5) zinc finger and BTB domain-containing protein 44 regulates DNA templates and transcription; (6) long-chain fatty acid-CoA ligase (ACSL) is associated with activation of long-chain fatty acids for both synthesis and degradation of cellular lipids; and (7) NOXA1 functions as an activator of NADPH oxidase 1 (NOX1), a superoxide-generating NADPH oxidase, in ROS generation.

The six unique proteins that were only expressed in the plasma of patients with coexisting T2DM/HT can be divided into four functional groups: structural proteins, catalytic enzymes, transcription, and cellular processes (Table 5). These proteins, as described in Uniprot, have the following functions: (1) MAP1A is mainly expressed in neurons, functions as a provider of microtubule-stabilizing activity, and interacts with other cellular components including filamentous actin and signaling proteins; (2) the function of HCG2022586, isoform CRAc, is still unknown; (3) transhydrogenase subunit alpha or NAD (P) is an enzyme that couples proton translocation across the inner mitochondrial membrane with the reversible transfer of hydride ion equivalents between NAD (H) and NADP (+); (4) transformation/transcription of domain-associated protein isoform 2 functions in histone acetyl-transferase activity; (5) zinc finger, the CCHC domain containing protein 13, functions as a DNA-binding motif in transcription; and (6) DGKB is an enzyme that phosphorylates DG and leads to the formation of phosphatidic acid (PA), which, in turn, regulates protein kinase C and phosphoinositide (PI) turnover activity.

### 3.4. Protein–Protein Interaction Network Analysis

**Figure 2:**
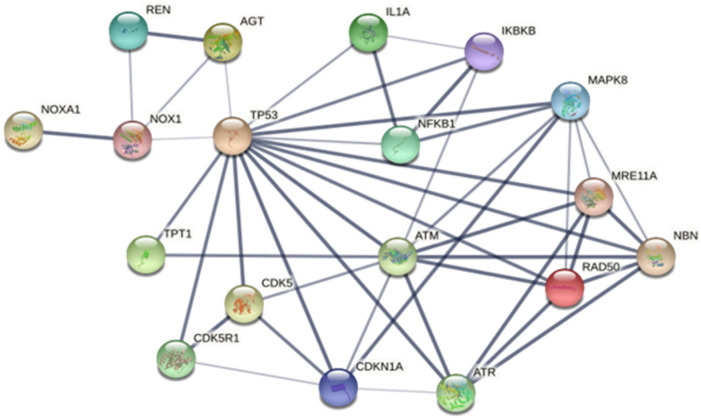
The protein–protein interaction of the unique proteins TPT1 and NBN found in T2DM using STRING 11.5. Proteins involved include DNA repair protein RAD50 (RAD50); double-strand break repair protein MRE11 (MRE11A); tumor protein p53 (TP53); serine protein kinase ATM (ATM); serine/threonine-protein kinase ATR (ATR); cyclin-dependent kinase inhibitor 1 (CDKN1A); cyclin-dependent-like kinase 5 (CDK5); mitogen-activated protein kinase 8 (MAPK8 or JNK1); inhibitor of nuclear factor kappa-B kinase subunit beta (IKBKB); nuclear factor NF-kappa-B (NFKB1); cyclin-dependent kinase 5 activator 1 (CDK5R1); NADPH oxidase activator 1 (NOXA1); NADPH oxidase 1 (NOX1); renin (REN); and angiotensinogen (AGT).

There were only six unique proteins expressed in T2DM plasma. Their functions were identified as responses to DNA damage. Among these proteins, protein–protein interaction between TPT1 and NBN was found using STRING 10 (Figure 2). Additionally, double-strand break (DSB) repair, genotoxic stresses, and DNA damage sensor proteins (MRE11, RAD50, TP53, ATM, ATR, and CDKN1A) induced inflammatory cytokines or DNA damage. Another group of proteins related to cellular stress (MAPK8 or JNK1, IKBKB or IKK^2^, and NFKB1) was involved in the death of neurons (CDK5R1 and CDK5) and the production of ROS proteins (NOX1 and NOXA1). Elevated blood pressure-related proteins (REN and AGT) demonstrated networks of interaction with TPT1 and NBN. Upon using STITCH 4.0, it was found that TPT1 and NBN interact with metformin but not with insulin (Figure S1). This confirms the correlation between TPT1 and NBN and T2DM, but not with type 1 diabetes.

**Figure 3:**
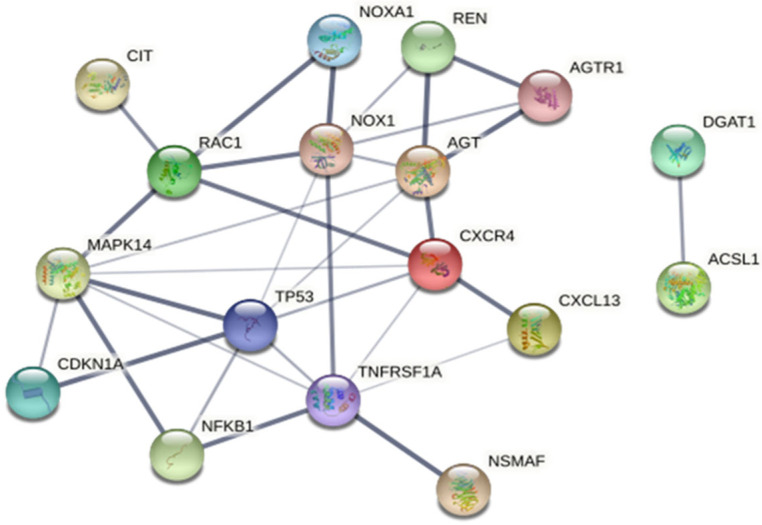
The protein interaction of the unique proteins NSMAF, CIT, and NOXA1 found in HT using STRING 11.5 included RAS-related C3 botulinum toxin substrate 1 (RAC1); NADPH oxidase 1 (NOX1); renin (REN); angiotensin II receptor type 1 (AGTR1); angiotensinogen (AGT); mitogen-activated protein kinase 14 (MAPK14); cyclin-dependent kinase inhibitor 1 (CDKN1A); tumor protein p53 (TP53); nuclear factor NF-kappa-B (NFKB1); and tumor necrosis factor receptor superfamily member 1A (TNFRSF1A); chemotactic for B-lymphocytes (CXCL13); C-X-C chemokine receptor type 4 (CXCR4); and unique ACSL1 proteins had interaction with diacylglycerol O-acyltransferase 1 (DGAT1).

The String (v.11) software revealed the presence of protein–protein interaction in three unique proteins: HT, NSMAF, CIT, and NOXA1. Twelve additional interacting proteins (TNFRSF1A, RAC1, NOX1, AGTR1, AGT, REN, CXCR4, CXCL13, CDKN1A, TP53 (p53), NFKB1, and MAPK14) were added to obtain a more comprehensive understanding of the interaction, as shown in Figure 3. RAS-related C3 botulinum toxin substrate 1 (RAC1) and TNFRSF1A are associated with protein function in the apoptotic pathway, and NOX1, REN, AGTR1, and AGT are proteins that function in developing HT by the renin–angiotensin II mechanism. CXCR4 contributes to the transduction of several signals by increasing intracellular calcium ion levels of CXCL13, also known as a chemotactic for B-lymphocytes. DGAT1 is involved in very-low-density lipoprotein (VLDL) assembly. STITCH 4.0 was also used to identify the interaction network between these three proteins and anti-hypertension drugs. The study revealed interactions between NSMAF, CIT, and NOXA1 and enalapril, a drug commonly used in HT treatment. These findings indicate an explicit relationship between HT and the three unique proteins of HT (Figure S2).

**Figure 4:**
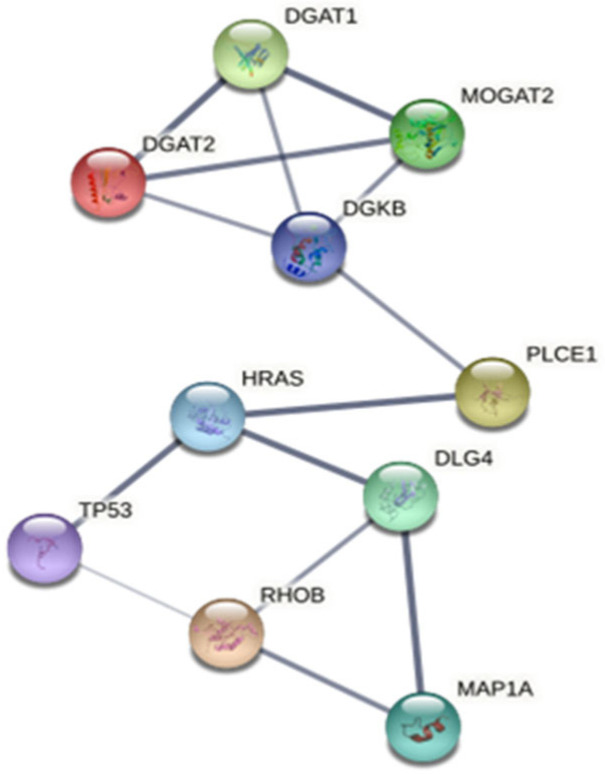
Protein interaction of the unique proteins MAP1A and DGKB found in T2DM/HT: rho-related GTP-binding protein RhoB (RhoB); discs large homolog 4 (DLG4); phospholipase C, epsilon 1 (PLCE1); cellular tumor antigen p53 (TP53); GTPase HRas (HRAS); diacylglycerol O-acyltransferase 2 (DGAT2); 2-acylglycerol O-acyltransferase 2 (MOGAT2); diacylglycerol O-acyltransferase 1 (DGAT1).

Six unique proteins were expressed only in the plasma of coexisting T2DM/HT patients. Upon bioinformatic analysis using String (v.11) software, it was observed that there were interactions between MAP1A and DGKB, as well as other proteins, as shown in Figure 4. P53 and RhoB were found to be associated with DNA damage responses. HRAS is involved in the activation of Ras protein signal transduction. DLG4 is involved in synaptic plasticity associated with the NMDA receptor. PLCE1 may play a role in cell survival, cell growth, actin organization, and T-cell activation. DGAT2, MOGAT2, and DGAT1 are involved in TG and VLDL synthesis. STITCH 4.0 was also used to identify the interaction network of MAP1A and DGKB with metformin and enalapril (Figure S3). These findings suggest that both MAP1A and DGKB are associated with T2DM and with HT.

## 4. Discussion

### 4.1. The Absence of NF-Kappa-B Inhibitor Epsilon and Bax Inhibitor 1 (BI-1) Inhibits Inflammation and Is Anti-Apoptotic

Healthy subjects showed upregulation of NF-kappa-B inhibitors epsilon and BI-1, indicating their association with inflammation inhibition and negative regulation of the apoptotic process. Interestingly, studies have shown that overexpression of BI-1 inhibits ROS accumulation under ER stress. BI-1 has also been shown to improve insulin sensitivity by increasing glycogen synthesis by augmenting GSK3 phosphorylation and by activating the JNK signaling pathway. In addition, BI-1 is involved with cytoprotective functions such as promotion of an antioxidant response via (nuclear factor erythroid 2-related factor 2) NRF2, increasing lysosome activity, and autophagy. [23,24]. Our study found that proteins that involved anti-inflammation and reduction of ROS were up-regulated in healthy and younger subjects compared to patients with T2DM, HT, and those with T2DM/HT. Inflammation and ROS play important roles in the pathogenesis of HT and diabetes. These findings suggest that younger individuals might express more of these two proteins that could prevent the development of insulin resistance and elevation of blood pressure. In particular, BI-1 has been reported to be a potential therapeutic target [23], indicating that these proteins might be appropriate for further study and investigation of new therapeutic targets for these conditions.

### 4.2. TPT1 and NBN Are Associated with DNA Damage, and NBN Is Related to the Aging Process and Age-Related Diseases Including Frailty via Activation of p53 in Hyperglycemic Conditions

The present study found that NBN and TPT1 proteins were up-regulated in T2DM and showed an association with DNA damage. Previous studies have described the correlation between hyperglycemia and DNA damage, despite not directly linking these proteins to diabetes. In diabetes, hyperglycemia can cause the generation of ROS, which can result in damage to cellular macromolecules, including DNA breaks [25,26]. Furthermore, the accumulation of DNA damage is linked to the aging process and the onset of age-related diseases. DNA damage plays a critical role in the impairment of glucose metabolism caused by oxidative stress and inflammation. DNA damage activates p53, which is involved with impaired insulin signaling via activation of the JNK/IKKβ pathways and induces beta cell dysfunction. NBN and TPT1 have been shown to interact with cellular response to DNA damage or DNA damage sensor proteins, as well as to DNA damage signaling like ATM, ATR MRE11, RAD50, CDKN1A (p21), and p5; impaired insulin signaling and inflammatory response proteins, e.g., JNK1, IKKβ, NFKB1, and IL1A; and ROS-generating mediated renin–angiotensin system proteins via p53. NBN and TPT1 are also associated with insulin resistance mediated by p53. Interestingly, studies have shown that NBN directly interacts with JNK1 and plays a major role in insulin resistance. Specifically, researchers found that NBN directly interacts with all these DNA damage-signaling proteins due to its membership in the MRN complex proteins (MRE11-RAD50-NBN), which significantly contribute to the DNA damage response by being among the first factors recruited into the DNA damage response (DDR) pathway. DDR triggers activation of the MRN complex, which can lead to activation of p53, a master regulator of the DDR pathway, e.g., DNA repair, senescence, and apoptosis, via both ATM and ATR activation. Persistent activation of the DDR triggers signaling cascades that can result in apoptosis or senescence to avoid replicating a damaged genome. This contributes to the onset of the aging process, which includes mitochondrial and metabolic dysfunction, altered proteostasis, inflammation, and the generation of high ROS levels. The p53 regulates the diverse outcomes of the DDR that are directly involved in promoting the transcription of DNA damage response genes, including cellular senescence. The chronic activation of p53 can lead to an accumulation of senescent cells. The senescence-associated secretory phenotype (SASP) includes pro-inflammatory cytokines, chemokines, and proteases. It changes the metabolism, shape, and secretory profile of senescent cells. Persistent activation of DDR-mediated p53 increases HMGB1 and SASP factors, e.g., IL1b, IL-6, IL-10, MCP-1, and TNF-α. DDR can also trigger NF-kB activation, a process primarily associated with aging. This activation is a major regulator of a large set of SASP components and also affects chronic inflammation and ROS generation. Thus, genotoxic stress is a key driver of aging, rather than simply something that occurs with aging and age-related conditions [27,28,29,30,31,32,33,34,35,36,37,38,39,40]. Interestingly, studies have also demonstrated the interaction of NBN and TPT1 with CDKN1A or p21, CDK5R1, CDK5, and IL1A-mediated p53. The p21 and p53 are well-established markers of senescence. The p53 activates p21 and contributes to senescence by enhancing the survival of senescent cells [37]. Three proteins, CDK5R1, CDK5, and IL1A, have been reported in plasma proteomic profiles of frail phenotypes associated with worsening frailty status over time in volunteers [38]. Diabetes is also linked to a higher risk of frailty. Moreover, studies have correlated the severity of frailty with decreased DNA repair capacity and increased DNA damage [39,40], suggesting a potential link between DNA damage in diabetes and the level of frailty.

**Figure 5:**
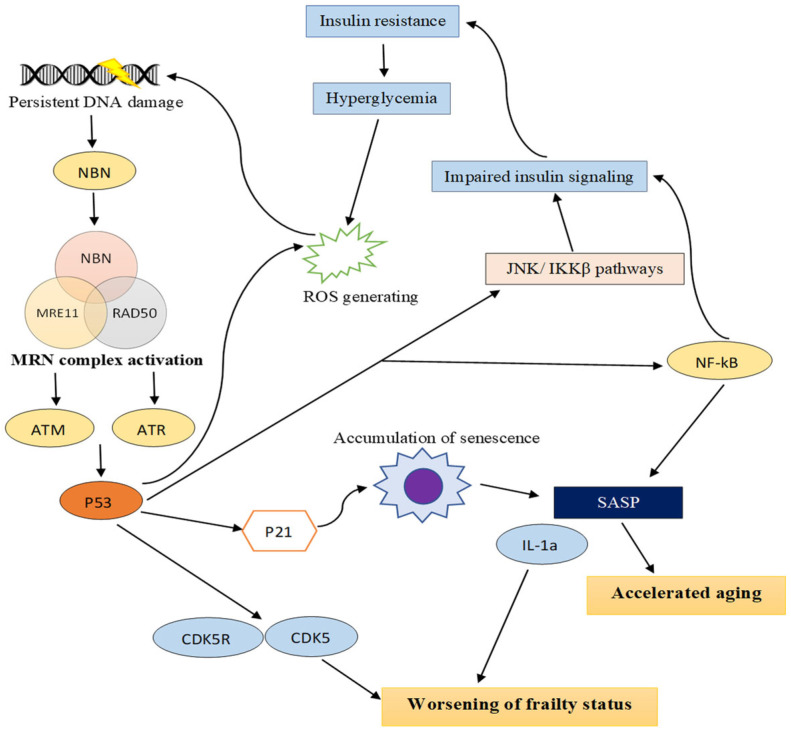
A proposed mechanism to explain how insulin resistance leads to accumulation of DDR, which is involved with accelerated aging, age-related frailty, and increased severity of frailty through activation of NBN-mediated p53.

According to our study, a possible mechanism that might explain insulin resistance is the fact that hyperglycemia promotes DDR accumulation, which then induces overexpression of NBN and leads to activation of p53. Chronic activation of p53 can activate p21, promote the survival of senescent cells, and increase the SASP factor. In addition, p53 stimulates activation of inflammatory signaling proteins, including NF-kB, IKKb, and IL1A, and increases ROS generation. Furthermore, DDR accumulation contributes to frailty status deterioration. We found that p53, p21, and ATM interact with proteins related to worsening of frailty status in the proteomics study. We also discovered that p53 is linked to proteins involved in ROS generation and elevated blood pressure. Thus, DNA damage that occurs in diabetic patients might be involved in accelerated aging and age-related conditions like increased frailty, as shown in Figure 5. NBN may play an important role in DNA damage-induced aging and complications of diabetes because this protein initiates DNA damage response signaling. Thus, NBN should be investigated in future studies of the relevance of the link between DM, aging, and DNA damage-related diseases (Figure 5).

### 4.3. ASCL1 Is Associated with Lipid Accumulation and Induced HT and ROS Production by the Ang II-Stimulated NOX1 and NOXA1 Activation Progression in Age-Related Diseases Such as HT, Frailty, and Cardiovascular Diseases (CVDs)

**Figure 6:**
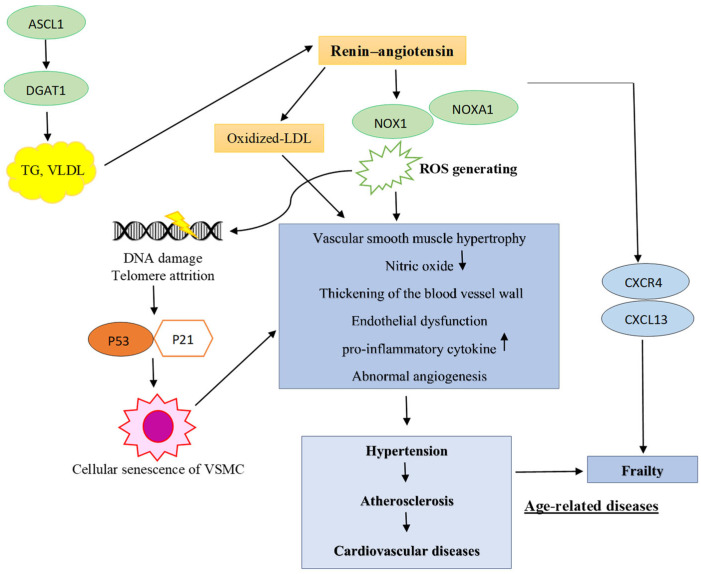
A proposed mechanism to explain the increased TG and VLDL synthesis and excessive ROS production by Ang II-stimulated NOX1-NOXA1 and ASCL1, which are associated with the progression of age-related diseases and conditions.

In the HT group, long-chain fatty acid-CoA ligase (ASCL1), which functions in the stimulation of triacylglycerol (TAG), cholesterol synthesis and the functioning of membrane phospholipids (PL), was found to have been up-regulated [41]. Our study also identified direct interaction between ASCL1 and DGAT1 (supplementary data). DGAT1 is involved in very-low-density lipoprotein (VLDL) assembly and TG synthesis. Previous reports have shown that lipid accumulation in blood vessels can enhance the expression of renin–angiotensin system (RAS) components. Activation of RAS also induces the accumulation of oxidized low-density lipoproteins (LDL) in blood vessels, and oxidized LDL can cause endothelial dysfunction and HT. On the other hand, activation of RAS can increase vasoconstriction and free radical production, which foster both HT and atherosclerosis [42,43]. We also found overexpression of NADPH oxidase activator 1 (NOXA1) in the plasma of HT patients. NOXA1 showed strong interaction with NOX1 and proteins associated with developing HT by the renin–angiotensin II mechanism (AGTR1, AGT, and REN). NOX1 played a critical role in inducing oxidative stress and subsequent cellular senescence, as well as age-related diseases such as HT and CVDs caused by angiotensin II (Ang II). NOXA1 also played an important role in generating ROS, because NOX1 oxidase activity requires cytosolic NOXA1. NOX1 expression occurs in endothelial cells, adventitial cells, and vascular smooth muscle cells (VSMCs) of human vascular and heart tissue, which are up-regulated by angiotensin II and pro-inflammatory cytokines in blood vessel walls under pathologic conditions. The overexpression of NOX1 can induce vascular smooth muscle hypertrophy via the angiotensin II mechanism. Ang II increases the production of ROS by stimulating the activation of NOX1, which is involved in the initiation and progression of HT through reduction of the bioavailability of nitric oxide, which serves as an important component in the development of increased blood pressure. NOX1 is also involved with the thickening of blood vessel walls, abnormal angiogenesis, atherosclerosis, and cellular senescence of VSMCs. It is known that too many ROS caused by Ang II activating NOX1 in vascular walls can speed up the aging process of cells. Oxidative stress is well established as a key regulator of age-induced endothelial dysfunction and the renin–angiotensin–aldosterone system (RAAS). We also found that NOXA1-NOX1 showed mild interaction with markers of senescence (p53 and/or p21-mediated p53). It has been reported that ROS generation by Ang II-stimulated NOX1 activation leads to telomere attrition and increased DNA damage, which, in turn, leads to activation of p53 and p21. The p21 has been identified as a SASP and a key factor in the progression of vascular diseases. Oxidative stress can lead to the buildup of senescent VSMCs in arteries, which is linked to abnormal vascular remodeling and stiffening of the arteries as people age. These senescent cells, through the SASP, may also contribute to the spread of senescence in the adventitia and endothelium, as well as the recruitment of inflammatory cells [44,45,46,47,48,49,50,51,52,53,54]. Interestingly, CXCL13, which is associated with frailty status [38], shows interaction with NOX1-Ang II proteins via CXCR4. The operation of the mechanism is shown in Figure 5. However, we found that NBN, which was only expressed in the T2DM groups, demonstrates a stronger correlation with p53, which in turn interacts with the worsening of frailty status marker proteins, compared to NOXA1. This suggests that T2DM subjects might be at greater risk for worsening of frailty status than hypertensive groups. In summary, excessive ROS production by Ang II-stimulated NOX1 activation induces inflammation, accumulation of senescent VSMCs, and abnormal angiogenesis, contributing to aging and to diseases prominent in the elderly, e.g., HT, atherosclerosis, diabetes, frailty, and CVDs. Studies have also reported overexpression of NOX1 in patients with CVDs, and NOX1 has been suggested as a therapeutic target for HT and CVDs through reduction of oxidative stress [48]. In addition, NOXA1 has also been reported to be up-regulated in aortas and atherosclerotic lesions, because NOXA1 is a critical component of VSMC NADPH oxidase [50]. We suggest that both NOX1 and NOXA1 may play important roles in the progression of age-related diseases and conditions, and they may be both therapeutic targets and monitoring biomarkers (Figure 6).

### 4.4. MAP1A Is Associated with Dementia via RhoB-p53 and DGKB, as Well as Being Involved in Dyslipidemia of HT in Patients with T2DM

Hyperinsulinemia and insulin resistance are directly linked to HT because insulin raises sympathetic activity in the autonomic nervous system and lowers prostaglandin production in adipose tissue. It also causes the tubules of the kidney to reabsorb more sodium. Together, these lead to increased muscle tonus and HT [55]. Furthermore, high insulin levels stimulate increased angiotensin II production [56]. We also discovered that MAP1A interacts with p53-mediated RhoB as an early DNA damage-inducible gene from genotoxic stress [57]. Genotoxic stress activates p53, which links RhoB to age-associated dementia [58]. Previous studies have reported both DM and HT are associated with a high risk of dementia [59]. However, there have been no studies reporting on the level of risk of dementia in patients with coexisting T2DM/HT. The current investigation discovered an upregulation of MAP1A in T2DM/HT patients, which could potentially link to the pathogenesis of DM and/or HT, as well as other complications stemming from the activation of DDR proteins and the development of dementia from DDR through p53-mediated RhoB activation. Diacylglycerol kinase beta (DG kinase) was also found in this group and showed interaction with MOGAT2, DGAT2, and DGAT2, which are directly involved in TG and VLDL synthesis. MOGAT2, or monoacylglycerol acyltransferase (MGAT), is primarily expressed in the small intestine and liver and plays a central role in the absorption of dietary fat in the small intestine, as well as in mediating rate-limiting intestinal TG absorption and catalyzing the synthesis of diacylglycerol (DG), a triacylglycerol precursor. Thus, MOGAT2 plays a role in diet-induced obesity and metabolic syndrome. There is a direct correlation between high triacylglycerol levels and obesity, hypertriglyceridemia, HT, and T2DM. The severity of metabolic syndrome strongly correlates with elevated triglyceride (TG) levels [60,61]. We found DGKB is directly correlated with these proteins and may lead to metabolic syndrome resulting from abnormal lipid metabolism. Furthermore, lipid accumulation in organs is associated with pathophysiological aging phenotypes, because increases in adipokine levels contribute to age-related diseases by modulating changes in systemic metabolism and inflammation. Studies have reported that across all age groups, total cholesterol, LDL, and TG increase with age [62,63]. This suggests that metabolic syndrome might lead to too much of the microtubule-associated protein 1a. This would raise the risk of dementia and other age-related diseases in HT patients with diabetes by activating p53 through RHOB. However, there have been few studies of MAP1A-related diseases. Future studies should be conducted to validate our finding regarding the association between MAP1A and complications of diabetes and HT. DGKB has also been shown to interact with p53 indirectly (via PLCE1 and HRAS). DGKB has also been shown to interact directly with induced obesity and abnormal lipid metabolism proteins, which play an important role in the pathogenesis of metabolic syndrome and age-related diseases resulting from dyslipidemia. Additionally, this group did not exhibit a significantly higher expression of proteins involved in DNA damage and/or ROS production compared to the DM and HT patient groups. This may indicate that T2DM/HT patients might not have a higher incidence of complications or other conditions, including CVD and frailty, than T2DM or HT patients.

This is a pilot study, and there are some limitations. The first limitation is that there were statistically significant differences in the mean age of the volunteers donating serum among the groups. The HT group was the oldest (66.87 years), followed by coexisting T2DM and HT (58.22 years), T2DM (57.38 years), and the healthy group (40.85), respectively. This is because the prevalence of T2DM and HT is higher in older age groups among the Thai population than in healthy individuals. From the reports on the prevalence of T2DM and HT in Thailand, it is evident that the prevalence of these conditions increases in individuals aged 45 and above [64]. Therefore, proteins identified in HT, T2DM, or T2DM/HT could potentially link to the aging process or age-related diseases like frailty and dementia. Second, some of the patients’ detailed clinical characterizations, which may affect their proteome, are insufficient and could influence the results of the analysis performed in this study. These include therapeutic management, the medical treatments of the patients with T2DM and HT, and the degree of disease severity. Third, the sample groups were relatively small. Additionally, pooling of samples was used to reduce control variability and to help overcome resource constraints [65]. The loss of data on biological variability could potentially reduce the study’s significance when using pooled samples. However, we also found proteins only expressed biological functions that are associated with conditions or diseases associated with T2DM and HT in previous studies. Further studies should be conducted using a larger sample to validate our study.

## 5. Conclusions

Our study found proteins associated with anti-inflammation and reduction of ROS were only expressed in healthy and younger subjects but were down-regulated in all other patients. This suggests that increasing age might increase the risk for metabolic syndromes by decreasing the expression of anti-inflammatory and anti-oxidation proteins. Conversely, decreased expression of these proteins might be associated with DM and HT, as well as other complications and age-related diseases, including accelerated aging. We also found that in the T2DM group, there was persistent activation of the DDRF due to overexpression of NBN as an initiator of regulator DNA damage signaling, which could lead to accumulation of senescent cells, increased ROS production, and chronic inflammation. This suggests that diabetes might be involved in accelerated aging, age-related diseases, and increased frailty. In the HT group, excessive ROS production by Ang II-stimulated NOX1 activation can lead to abnormal angiogenesis, atherosclerosis, cellular senescence of VSMC, and endothelial dysfunction. Together, these factors can contribute to pathogenesis, including age-related conditions such as HT, atherosclerosis, and frailty. For coexisting HT and T2DM patients, we found only overexpression of MAP1A to be involved with dementia through activation of p53-RhoB and DGKB. This overexpression may be linked to induced obesity and abnormal lipid metabolism, conditions that can contribute to pathogenesis, such as dyslipidemia, and other complications in patients with both HT and T2DM.

An analytical study conducted on the interaction between these proteomics and STRING revealed an association with cellular senescence and geriatric syndromes. Further study of these relationships is needed. In addition, our findings need to be validated in future studies. These unique proteins may be expressed in only a small subset of the subjects, making them potential candidates for protein biomarkers that will require further investigation.

## Figures and Tables

**Table 1:** Clinical characteristics of study subjects and statistical analysis.

Characteristic	Healthy Subjects (*n* = 14)	T2DM (*n* = 13)	HT (*n* = 16)	Coexisting T2DM and HT (*n* = 18)	*p*-Value
Gender (Male: Female)	2:12	7:6	6:10	6:12	0.196
Age (years)					
Mean ± SD	40.85 ± 7.12	57.38 ± 6.03	66.87 ± 10.09	58.22 ± 10.65	<0.01
Range	21–51	48–68	46–79	39–80	
BW (kg)					
Mean ± SD	55.00 ± 7.81	57.92 ± 8,52	55.68 ± 9.94	65.22 ± 11.52	0.013
FPS (mg/dL)					
Mean ± SD	88.86 ± 9.4	150.53 ± 46.99	99.50 ± 8.02	156.33 ± 29.80	<0.01
SBP(mmHg)					
Mean ± SD	115.92 ± 6.03	117.07 ± 6.04	145.62 ± 14.60	144.22 ± 11.20	<0.01
DBP(mmHg)					
Mean ± SD	69.07 ± 8.61	73.00 ± 4.89	82.00 ± 9.23	76.72 ± 8.74	<0.01

T2DM: type 2 diabetes; HT: hypertension; BW: body weight; FPS: fasting plasma sugar; SBP: systolic blood pressure; DBP: diastolic blood pressure. *p* value < 0.05 considered as significant.

**Table 2:** Identification and functional classification of eight unique proteins present in plasma of healthy controls, as determined by Uniprot.

Protein	Accession No.	Peptide Sequence	Functional Category	Specific Function
(1) NF-kappa-B inhibitor epsilon	gi 71274109	GGGGAIR	Transcription	Inhibits DNA-binding of NF-kappa-B
(2) Synaptonemal complex protein 3	gi 24233580	MVSSGKK	Cellular processes	Required for normal meiosis during spermatogenesis and male fertility
(3) HCG2041603	gi 119574203	GVEFGK	-	-
(4) Protein SZT2	gi 153217507	MGPSMGVSR	ROS-generating	Association with superoxide dismutase activity and neuroprotective effect of peroxisomes
(5) RanBP2 (Ran-binding protein 2)	gi 1009337	GKQDFLK	Signaling protein	Transport factor (Ran-GTP, karyopherin)-mediated protein
(6) Diphthine methylester synthase	gi 12005667	GIHNASIMNAEAAGGYR	Metabolism	Involved in the pathway peptidyl-diphthamide biosynthesis
(7) Diacylglycerol kinase zeta	gi 2183038	VVCDGMDLTPK	Metabolism	Converts diacylglycerol/DAG into phosphatidic acid/phosphatidate/PA
(8) Bax inhibitor 1 isoform 2	gi 148746181	SHSSVTR	Signaling protein	Negative regulation of apoptotic process

**Table 3:** Identification and functional classification of six unique proteins present in plasma of T2DM, as determined using Uniprot.

Protein Name	Accession No.	Peptide Sequence	Functional Category	Specific Function
(1) HCG32984	gi 119610937	ALESQLEARAAANAELR	-	-
(2) Huntingtin-interacting protein 1-related protein	gi 48762942	FCHVLHK	Binding protein	Endocytic machinery to actin cytoskeleton
(3) Nibrin isoform X1	gi 119574203	GVEFGK	Binding protein	Cellular response to DNA damage
(4) Translationally controlled tumor protein (TCTP)	gi 25058985	DYMKS	Signaling protein	Negative regulation of intrinsic apoptotic signaling pathway in response to DNA damage
(5) Immunoglobulin alpha heavy chain variable region	gi 62871116	WVHRPGXGSQGAFFP	-	-
(6) Immunoglobulin gamma 1 heavy chain variable region	gi 304562876	LSCAASGFSFSIYWMTWVR	-	-

**Table 4:** Identification and functional classification of expressions of seven unique proteins in plasma of HT patients, from Uniprot.

Protein Name	Accession No.	Peptide Sequence	Functional Category	Specific Function
(1) SH3 and cysteine-rich domain-containing protein	gi 4507247	EDGVDGLPK	Binding protein	Cellular response to heat
(2) Protein FAN isoform 1	gi 31543297	ANHILHK	Signaling protein	TNF-α-induced cell signaling
(3) Citron (rho-interacting, serine/threonine kinase 21), isoform CRA_b	gi 119618562	ATKCAVCLDTVHFGR	Binding protein	Regulation of cytokinesis
(4) Probable ribonuclease 11 precursor	gi 21687041	NTSLSMSK	Binding protein	Nucleic acid phosphodiester bond hydrolysis
(5) Zinc finger and BTB domain-containing protein 44	gi 74760158	VQDKIFR	Transcription	Regulation of transcription, DNA-templated
(6) Long-chain fatty acid-CoA ligase	gi 489945352	LSTMMDIPLSITR	Metabolism	Triacylglycerol synthesis and beta-oxidation
(7) NADPH oxidase activator 1 isoform 2	gi 366039970	HLEPVDFLGK	ROS-generating	Donate an electron from NADPH to O_2_ to produce superoxide (O_2-_)

**Table 5:** Identification and functional classification of six unique proteins present in plasma of patients with coexisting T2DM/HT, identified by Uniprot.

Protein Name	Accession No.	Peptide Sequence	Functional Category	Specific Function
(1) Microtubule-associated protein 1a	gi 1790878	EDGVDGLPK	Structural protein	The filamentous cross-bridging between microtubules and other skeletal elements
(2) HCG2022586, isoform CRA_c	gi119599705	ANHILHK	-	-
(3) NAD(P) transhydrogenase subunit alpha	gi497804994	ATKCAVCLDTVHFGR	Catalytic enzyme	Catalyzes conversion of NADPH and NAD^+^ to NADP^+^ and NADH
(4) Transformation/transcription domain-associated protein isoform 2	gi4507691	NTSLSMSK	Transcription	Histone acetyltransferase activity (HAT)
(5) Zinc finger, CCHC domain containing 13, isoform CRA_a	gi119619052	VQDKIFR	Transcription	DNA-binding motif in transcription
(6) Diacylglycerol kinase beta isoform 1	gi22027632	LAQCSCVVIRTSK	Lipid metabolism	Converts diacylglycerol/DAG into phosphatidic acid/phosphatidate/PA

## Data Availability

Data are available on request.

## Supplementary Materials

The following supporting information can be downloaded at: https://www.mdpi.com/article/10.3390/life14101269/s1; Figure S1: Interactions of the two unique proteins (*TPT1 and *NBN) found in type 2 diabetes with metformin and insulin by using STITCH 4.0: Sirtuin 1 (SIRT1) and tumor protein p53 (TP53). Figure S2: Interaction of the unique proteins found in hypertension (NSMAF, CIT, and NOXA1) with enalapril (CAS 76095-16-4). RAS-related C3 botulinum toxin substrate 1 (RAC1); NADPH oxidase 1 (NOX1); renin (REN); angiotensin II receptor type 1 (AGTR1); angiotensinogen (AGT); tumor necrosis factor receptor superfamily, member 1A (TNFRSF1A); angiotensin I converting enzyme 1 (ACE); and Rho GDP dissociation inhibitor (GDI) alpha (ARHGDIA). Figure S3: Interaction of the unique proteins (MAP1A and DGKB) found in coexisting type 2 diabetes and hypertension with metformin and enalapril. Nuclear factor of kappa light polypeptide gene enhancer in B-cell inhibitor (NFKBIA); protein kinase C, epsilon (PRKCE); glutamate receptor, ionotropic N-methyl D-aspartate 2B (GRIN2B); angiotensin I converting enzyme (ACE); discs large homolog 4 (DLG4); protein kinase C, beta (PRKCB); V-Ha-RAS Harvey rat sarcoma viral oncogene homolog (HRAS); ubiquitin C (UBC); inhibitor of kappa light polypeptide gene enhancer in B-cells, kinase gamma (IKBKG); and beta-transducin repeat containing (BTRC).
