# Supplementary material for: Plasma Proteomics of Type 2 Diabetes, Hypertension, and Co-Existing Diabetes/Hypertension in Thai Adults
Source: Life (Basel). 2024 Oct 5;14(10):1269. doi: 10.3390/life14101269 (PMC11509282; doi:10.3390/life14101269)
Supplement: Supplementary file 1 [file life-14-01269-s001.zip › life-3131462-supplementary.pdf]

Supplementary Materials

Protein–chemical interaction by STITCH 4.0

1. Interaction between two unique proteins (TPT1 and NBN) and metformin

Upon using STITCH 4.0, it was found that TPT1 and NBN have interactions with metformin and apoptotic regulation proteins including SIRT1 and TP53 but not with insulin (Figure S1). Metformin decreases hyperglycemia primarily by suppressing glucose production by the liver via hepatic gluconeogenesis. Metformin also increases insulin sensitivity, enhances peripheral glucose uptake by inducing the phosphorylation of the GLUT4 enhancer factor, decreases insulin-induced suppression of fatty acid oxidation [58], and decreases the absorption of glucose from the gastrointestinal tract. Increased peripheral use of glucose may be due to improved insulin binding to insulin receptors.

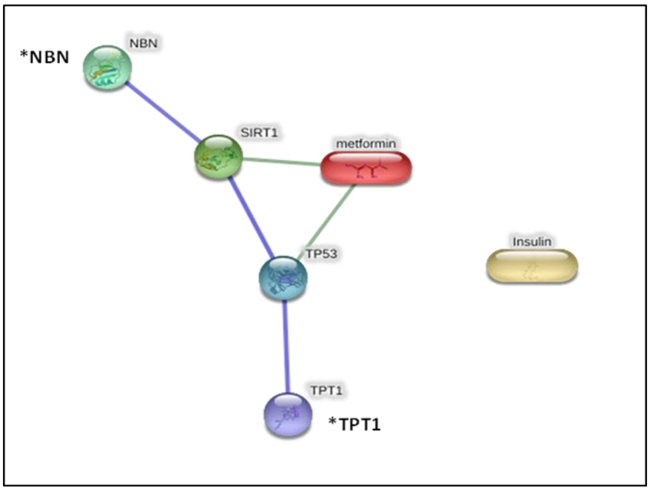

Figure S1. Interactions of the two unique proteins (*TPT1 and *NBN) found in type 2 diabetes with metformin and insulin by using STITCH 4.0: Sirtuin 1 (SIRT1) and tumor protein p53 (TP53).

2. Interaction between three unique proteins (NSMAF, CIT, and NOXA1) and Enalapril

STITCH 4.0 was also used to find out the interaction network between these three proteins including NSMAF, CIT, and NOXA1 and anti-hypertension drugs. There are many types of anti-hypertension drugs or those that can act like enalapril. In this study, enalapril could find most of the interactions with the unique proteins of hypertension. Enalapril is used in the treatment of hypertension as it can inhibit the angiotensin-converting enzyme (ACE) so that angiotensin I cannot convert to angiotensin II [59]. By using STITCH 4.0, it was found that NSMAF, CIT, and NOXA1 have interactions with enalapril; RAS-related C3 botulinum toxin substrate 1 (RAC1); NADPH oxidase 1 (NOX1); renin (REN); angiotensin II receptor type 1 (AGTR1); angiotensinogen (AGT); tumor necrosis factor receptor superfamily, member 1A (TNFRSF1A); angiotensin I converting enzyme 1 (ACE); and Rho GDP dissociation inhibitor (GDI) alpha (ARHGDIA) (Figure S2). These findings suggest that the three unique proteins of hypertension related exactly with hypertension.

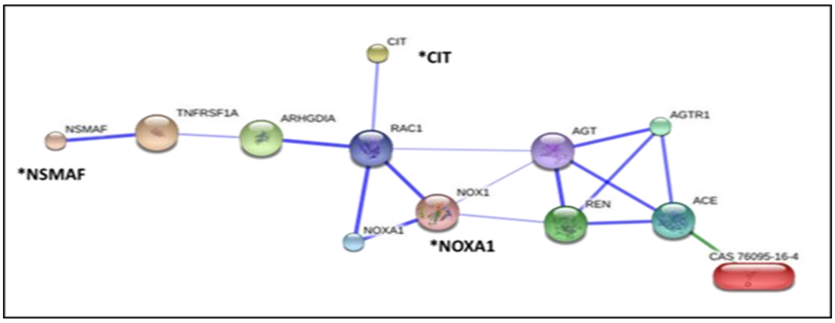

Figure S2. Interaction of the unique proteins found in hypertension (NSMAF, CIT, and NOXA1) with enalapril (CAS 76095-16-4). RAS-related C3 botulinum toxin substrate 1 (RAC1); NADPH oxidase 1 (NOX1); renin (REN); angiotensin II receptor type 1 (AGTR1); angiotensinogen (AGT); tumor necrosis factor receptor superfamily, member 1A (TNFRSF1A); angiotensin I converting enzyme 1 (ACE); and Rho GDP dissociation inhibitor (GDI) alpha (ARHGDIA).

3. Interaction between two unique proteins (MAP1A and DGKB) and metformin and enalapril

To ensure that these two unique proteins of coexisting type 2 diabetes and hypertension (MAP1A and DGKB) are involved in type 2 diabetes and hypertension, STITCH 4.0 was used to find out the interaction network between these two proteins and metformin and enalapril. Upon using STITCH 4.0, it was found that these proteins also showed interaction with metformin, enalapril, and other interaction proteins including NFKBIA, PRKCE, GRIN2B, ACE, DLG4, PRKCB, HRAS, UBC, IKBKG, and BTRC (Figure S3).

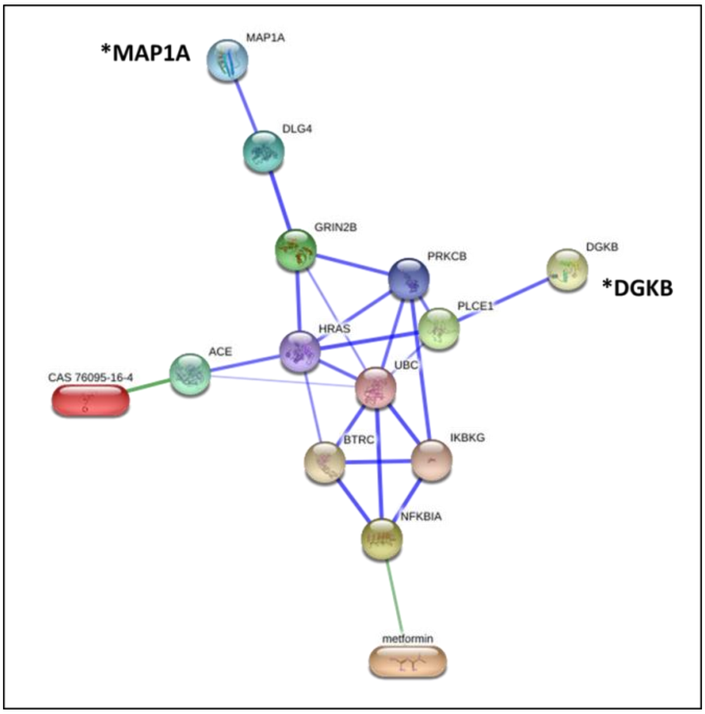

Figure S3. Interaction of the unique proteins (MAP1A and DGKB) found in coexisting type 2 diabetes and hypertension with metformin and enalapril. Nuclear factor of kappa light polypeptide gene enhancer in B-cell inhibitor (NFKBIA); protein kinase C, epsilon (PRKCE); glutamate receptor, ionotropic N-methyl D-aspartate 2B (GRIN2B); angiotensin I converting enzyme (ACE); discs large homolog 4 (DLG4); protein kinase C, beta (PRKCB); V-Ha-RAS Harvey rat sarcoma viral oncogene homolog (HRAS); ubiquitin C (UBC); inhibitor of kappa light polypeptide gene enhancer in B-cells, kinase gamma (IKBKG); and beta-transducin repeat containing (BTRC).
